# Biofilm Development by *Mycobacterium avium* Complex Clinical Isolates: Effect of Clarithromycin in Ultrastructure

**DOI:** 10.3390/antibiotics13030263

**Published:** 2024-03-16

**Authors:** Arij Akir, Abrar Senhaji-Kacha, Maria Carmen Muñoz-Egea, Jaime Esteban, John Jairo Aguilera-Correa

**Affiliations:** 1 Department of Clinical Microbiology, IIS-Fundación Jiménez Díaz, Universidad Autónoma de Madrid, 28040 Madrid, Spain; arij_akir@yahoo.co.uk (A.A.); abrarsen7@gmail.com (A.S.-K.); mcme86@gmail.com (M.C.M.-E.); john_j2a@hotmail.com (J.J.A.-C.); 2 CIBERINFEC-CIBER de Enfermedades Infecciosas, 28029 Madrid, Spain

**Keywords:** Biofilm, *Mycobacterium avium* complex, *Mycobacterium avium*, *Mycobacterium intracellulare*, *Mycobacterium chimaera*, clarithromycin, macrolides

## Abstract

Background: The *Mycobacterium avium* complex includes the commonest non-tuberculous mycobacteria associated with human infections. These infections have been associated with the production of biofilms in many cases, but there are only a few studies about biofilms produced by the species included in this group. Methods: Three collection strains (*M. avium* ATCC25291, *M. intracellulare* ATCC13950, and *M. chimaera* DSM756), three clinically significant strains (647, 657, and 655), and three clinically non-significant ones (717, 505, and 575) of each species were included. The clinical significance of the clinical isolates was established according to the internationally accepted criteria. The biofilm ultrastructure was studied by Confocal-Laser Scanning Microscopy by using BacLight Live–Dead and Nile Red stains. The viability, covered surface, height, and relative autofluorescence were measured in several images/strain. The effect of clarithromycin was studied by using the technique described by Muñoz-Egea et al. with modifications regarding incubation time. The study included clarithromycin in the culture medium at a concentration achievable in the lungs (11.3 mg/L), using one row of wells as the control without antibiotics. The bacterial viability inside the biofilm is expressed as a percentage of viable cells. The differences between the different parameters of the biofilm ultrastructure were analyzed by using the Kruskal–Wallis test. The correlation between bacterial viability in the biofilm and treatment time was evaluated by using Spearman’s rank correlation coefficient (ρ). Results: The strains showed differences between them with all the studied parameters, but neither a species-specific pattern nor a clinical-significance-specific pattern were detected. For the effect of clarithromycin, the viability of the bacteria contained in the biofilm was inversely proportional to the exposure time of the biofilm (ρ > −0.3; *p*-value < 0.05), excluding two *M. chimaera* strains (*M. chimaera* DSM756 and 575), which showed a weak positive correlation with treatment time (0.2 < ρ < 0.39; *p*-value < 0.05). Curiously, despite a clarithromycin treatment of 216 h, the percentage of the biofilm viability of the strains evaluated here was not less than 40% at best (*M. avium* 717). Conclusions: All the *M. avium* complex strains studied can form biofilm in vitro, but the ultrastructural characteristics between them suggest that these are strain-specific characteristics unrelated to the species or the clinical significance. The clarithromycin effect on MAC species is biofilm-age/time-of-treatment-dependent and appears to be strain-specific while being independent of the clinical significance of the strain.

## 1. Introduction

Non-tuberculous mycobacteria (NTM) include many species of the genus *Mycobacterium* that have been known since the first years after the discovery of *M. tuberculosis* [1]. Among these organisms, the *M. avium* complex (MAC) includes several species of NTM [2], especially three: *M. avium*, *M. intracellulare*, and *M. chimaera*, which are considered the commonest NTM isolated throughout the world [3]. In a multicenter and multinational study by Hoefsloot et al., MAC was the commonest NTM isolated in almost all countries [3].

MAC species are also important because they are a cause of human infection, especially respiratory infections among patients with underlying diseases at the present moment [2,4]. It can also cause epidemic outbreaks, like those caused by *M. chimaera* related to the heating–cooling machines used in cardiac surgery [5,6]. These infections are currently considered biofilm-related infections, and this fact is of clinical importance because of the higher resistance against antibiotics of sessile bacteria compared with planktonic ones. However, despite these facts, there are only a few in vitro studies regarding biofilm formation by MAC [7,8,9,10,11,12], and most of them used only collection strains. Here we report a study about biofilm formation by the three most important species of MAC and the effect of clarithromycin, an antibiotic commonly used in these infections, on them, using not only collection strains but also clinical isolates of all of them. 

## 2. Material and Methods

### 2.1. Strains

During this study, the following strains were used: *M. avium* ATCC 25291 (type strain), *M. intracellulare* ATCC 13950 (type strain), and *M. chimaera* DSM 756 (type strain). Clinical strains of all these species were also included: *M. avium* (717 and 647), *M. intracellulare* (505 and 657), and *M. chimaera* (575 and 655). The clinical significance of the clinical isolates was established according to the ATS-ERS-IDSA-ESCMID criteria [4]. All the strains were kept frozen at −80 °C. Before the experiments were performed, all the strains were defrosted, inoculated onto Middlebrook 7H10 agar plates, incubated at 37 °C for 15 days, and checked for purity. 

### 2.2. Biofilm Development

The studies of biofilm growth were performed by using a modification of the technique described by Muñoz-Egea et al. [13,14]. We used 2 × 4 well plates with an uncoated hydrophobic surface (Ibidy GmbH, Martinsried, Germany) with a prolongation of the times for the study of biofilm development to 48, 120, 192, and 264 h. Four wells were stained with a Nile Red© stain (Sigma-Aldrich Co., St. Louis, MO, USA), and the other four wells were stained with a Live/Dead BacLight© stain (Invitrogen, Waltham, MA, USA) according to the instructions provided by the manufacturers. Leica DM IRB microscopy (Leica, Wetzlar, Germany) was used for Confocal Laser Scanning Microscopy (CLSM). Each time we evaluated 4 parameters: the % of the covered surface, thickness, viability, and presence of autofluorescence. The last parameter was analyzed according to the following formulation: (% autofluorescence of covered surface/% Nile Red covered surface) × 100. All the experiments were performed in triplicate. 

### 2.3. Effect of Clarithromycin on Biofilm Ultrastructure

The same study described above was performed by including clarithromycin in the culture medium at a concentration of 11.3 mg/L at different time points, using one row of wells as the control without antibiotics. This concentration is the concentration of clarithromycin reached in the lungs when a patient is receiving this treatment [15]. After the treatment, each well was stained with a Live/Dead BacLight© stain. All the strains were tested in triplicate.

### 2.4. Clarithromycin Susceptibility of Planktonic and Sessile Bacteria

We studied the Minimum Inhibitory Concentration (MIC) by using the recommended broth microdilution method [16] with one modification: In brief, two-fold dilutions of clarithromycin concentrations ranging from 0.5 to 512 µg/mL were added to cation-adjusted Müller–Hinton broth (Sigma Aldrich, St. Louis, MI, USA) (CAMHB) supplemented with oleic, albumin, dextrose, and catalase (OADC) to a final volume of 100 µL per well. A 1-MacFarland suspension of bacteria was performed in CAMHB, and 100 microliters of this suspension was added to a Costar 96-well round-bottom polypropylene plate (Corning Inc., Corning, NY, USA) followed by static incubation at 37 °C and 5% CO_2_ for at least 10 days. After incubation, MIC was determined by the naked eye as the well with the lowest concentration of clarithromycin where no bacterial growth was observed. We determined the Minimum Bactericidal Concentration (MBC) by using the flash microbiocide method [17]. Briefly, 20 µL of each well from the MIC 96-well plate were mixed after 24 h incubation with 180 µL of CAMHB supplemented with OADC in a new 96-well plate. This plate was then incubated statically at 37 °C and 5% CO_2_ for 10 days. After incubation, the MBC was determined by the naked eye as the well with the lowest concentration of clarithromycin where no bacterial growth was observed. The experiments were performed in triplicates.

The Minimal Biofilm Inhibitory Concentrations (MBICs) and minimal biofilm eradication concentrations were determined. For the study of the MBIC, biofilms were developed on untreated 96-well flat-bottom plates (Thermo Fisher Scientific, Waltham, MA, USA). A bacterial suspension of 1 McFarland in sterile tap water of each strain was performed, and 150 µL of the suspension was inoculated in each well for 3 h at 37 °C and 5% CO_2_ for the adherence of the bacterial cells After incubation, each well was washed with 150 µL of sterile tap water, and 150 µL of Middlebrook 7H9 supplemented with OADC were inoculated in each well. The plate was then statically incubated at 37 °C and 5% CO_2_ for 7 days. After biofilm growth, each well was washed with 150 µL of sterile phosphate buffer saline and filled with 200 µL of CAMHB supplemented with OADC containing concentrations of clarithromycin, ranging from 0.5 to 512 µg/mL. The plate was statically incubated at 37 °C and 5% CO_2_ for another 10 days. After incubation, the MBIC was determined by the naked eye as the well with the lowest concentration of clarithromycin where no bacterial planktonic growth was observed. All the experiments were performed in triplicates.

### 2.5. Statistical Analysis

Stata Statistical Software Release 11.0 (StataCorp 2009) was used for statistical studies. The Shapiro–Wilk test was used to evaluate the normality of the data. The descriptive data are cited as the median and interquartile range if a non-normal distribution was calculated. A non-parametric Wilcoxon test was used to compare the differences between the different parameters evaluated in the biofilm structural study of two groups, while a non-parametric Kruskal–Wallis test was used to compare more than two groups. The correlation between the bacterial viability in the biofilm and the treatment time was evaluated by using Spearman’s rank correlation coefficient (ρ). For absolute values of ρ, 0–0.19 is considered as very weak, 0.2–0.39 as weak, 0.40–0.59 as moderate, 0.6–0.79 as strong, and 0.8–1 as a very strong correlation. 

## 3. Results

### 3.1. Strains

The clinical significance of the clinical isolates was established according to the ATS-ERS-IDSA-ESCMID criteria, whereby the strains *M. avium* 647, *M. intracellulare* 657, and *M. chimaera* 655 were considered clinically significant. The clinical strains *M. avium* 717, *M. intracellulare* 505, and *M. chimaera* 575 were found to be non-clinically significant. 

### 3.2. Biofilm Forming Studies

**Figure 1:**
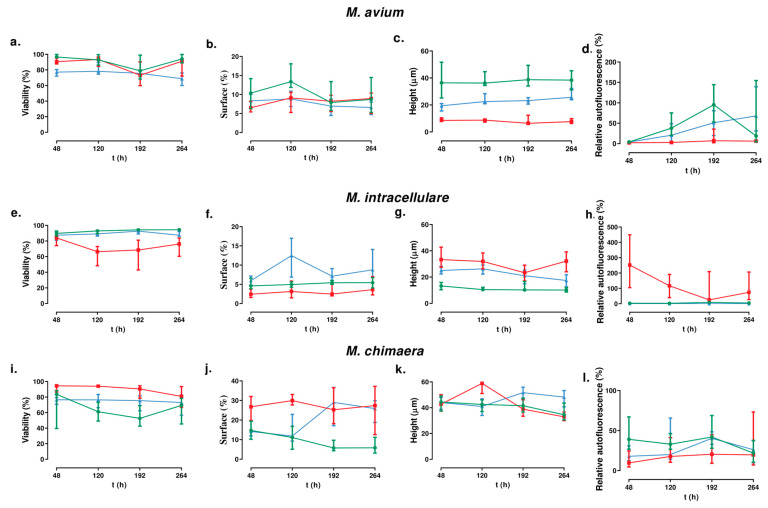
Biofilm study for viability (**a**,**e**,**i**), surface (**b**,**f**,**j**), height (**c**,**g**,**k**), and relative autofluorescence (**d**,**h**,**l**). Green, blue, and red represent reference strains, non-clinically significant strains, and clinically significant strains of each strain, respectively. The error bars represent the interquartile range.

**Figure 2:**
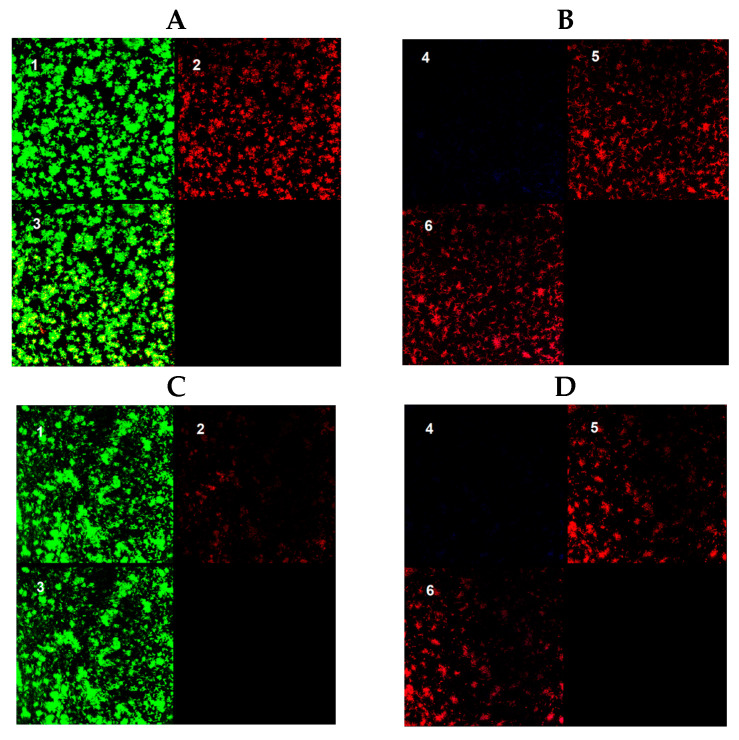
Images obtained for a 48 h biofilm of *M. avium* (**A**,**B**) and *M. intracellulare* (**C**,**D**). (**A**,**C**): BacLight© Live–Dead stain. (**B**,**D**): Autofluorescence and Nile Red stain. 1: Live bacteria; 2: dead bacteria; 3: merged Live–Dead bacteria; 4: autofluorescence; 5: Nile Red stained bacteria; 6: merged autofluorescence–Nile Red stained bacteria.

The results obtained are represented in Figure 1. Examples of the images are shown in Figure 2.

The time evaluation of the viability for the *M. avium* strains showed that no differences can be detected for the reference strain (*p*-value = 0.1212), but there are differences for the clinical strains (*p*-values = 0.0052 and 0.0018 for strains 647 and 717, respectively). For *M. intracellulare*, the variation was observed for the reference strain (*p*-value < 0.0001) and strain 657 (*p*-value = 0.0248) but not for strain 505. For *M. chimaera,* the variation showed similar characteristics as *M. intracellulare* (*p*-values = 0.0.0227 for the reference strain and 0.0003 for strain 655, but no significance for strain 575).

For the covered surface, we observed differences for the *M. avium* reference strain (*p*-value = 0.0202) and strain 717 (*p*-value = 0.0.0001), for *M. intracellulare* strains 657 (*p*-value = 0.0234) and 505 (*p*-value = 0.0042), and for the *M. chimaera* reference strain (*p*-value < 0.0001) and strain 575 (*p*-value = 0.0426). 

Differences in the height evolution with time were observed for the *M. avium* clinical strain (*p*-value = 0.0913 and <0.0001 for an increase in height for strains 647 and 717, respectively), for a height decrease in the *M. intracellulare* reference strain and strain 505 (*p* < 0.0001 for both of them), and an increase in *M. chimaera* strain 575 (*p*-value = 0.0001) but a decrease in the *M. chimaera* reference strain (*p*-value = 0.0019) and strain 655 (*p*-value < 0.0001). No differences in height were detected for the other strains.

Differences in autofluorescence evolution were also detected for all the strains of *M. avium* (*p*-values = 0.0004 for the reference strain and <0.0001 for both clinical strains), for the *M. intracellulare* reference strain (increase, *p*-value = 0.0101) and strain 655 (decrease, *p*-value = 0.0002), and for the *M. chimaera* reference strain (decrease, *p*-value = 0.0426) and strain 655 (increase, *p*- value = 0.0007).

We also analyzed the intraspecies variations, with the following results:

#### 3.2.1. For *M. avium*

At 48 h: The viability of the three strains was significantly different (*p*-value = 0.001 for the Kruskal–Wallis test). In descending order: the reference strains, strain 647, and strain 717. Each strain was statistically different from the others (*p*-value < 0.05 for the Wilcoxon test). The surface area covered by the reference and 717 strains was similar (*p*-value = 0.1345 for the Wilcoxon test). The surface area covered by strain 647 was significantly less than the reference and 717 strains (*p*-value < 0.05 for the Wilcoxon test). The autofluorescence of the three strains was significantly different (*p*-value = 0.0003 for the Kruskal–Wallis test). In descending order: strain 647, the reference strain, and strain 717. There were no significant differences between the reference and CON strains (*p*-value = 0.7092 for the Wilcoxon test). The biofilm height of the three strains was significantly different (*p*-value = 0.0001 for the Kruskal–Wallis test). In descending order: the reference strain, strain 647, and strain 717.

At 120 h: The viability of the three strains was significantly different (*p*-value = 0.0001 for the Kruskal–Wallis test). The viability of strain 717 was significantly lower than the rest of the strains (*p*-value < 0.001). There were no significant differences between the reference strain and strain 647 (*p*-value = 0.3472). The surface area covered by the reference and 707 strains was similar (*p*-value = 0.0001 for the Wilcoxon test). The surface area covered by the reference strain was significantly greater than that of the clinical strains (*p*-value < 0.00001 for the Wilcoxon test). There were no significant differences between strain 717 and strain 647 (*p*-value = 0.4688). The autofluorescence of the three strains was significantly different (*p*-value = 0.0001 for the Kruskal–Wallis test). In descending order, the highest was the reference strain, followed by strain 717 and strain 647. The biofilm height of the three strains was significantly different (*p*-value = 0.0001 for the Kruskal–Wallis test). In descending order, it was highest for the reference strain, followed by strain 717 and strain 647.

At 192 h: The viability of the three strains was similar (*p*-value = 0.1995 for the Kruskal–Wallis test), as well as the covered surface area of the three strains (*p*-value = 0.2198 for the Kruskal–Wallis test). The autofluorescence of the three strains was significantly different (*p*-value = 0.0001 for the Kruskal–Wallis test). In descending order, the highest value was detected for the reference strain, followed by strain 717 and strain 647, with each strain being statistically different from the others (*p*-value < 0.05 for the Wilcoxon test). The biofilm height of the three strains was significantly different (*p*-value = 0.0001 for the Kruskal–Wallis test). In descending order, it was highest for the reference strain, followed by strain 717 and strain 647. Again, the value obtained for each strain was statistically different from the others (*p*-value < 0.05 for the Wilcoxon test). 

At 264 h: The viability of the three strains was significantly different (*p*-value = 0.0001 for the Kruskal–Wallis test). In descending order: the reference strain, strain 647, and strain 717. Each strain was statistically different from the others (*p*-value < 0.05 for the Wilcoxon test). The surface area covered by the reference and 717 strains was similar (*p*-value = 0.0152 for the Wilcoxon test). The surface area covered by strain 717 was significantly greater than the 647 and the reference strains. (*p*-value < 0.05 for the Wilcoxon test). There were no significant differences between the reference strain and strain 647 (*p*-value = 0.5642). The biofilm height of the three strains was significantly different (*p*-value = 0.0001 for the Kruskal–Wallis test). In descending order: the reference strain, strain 717, and strain 647. The autofluorescence of the three strains was significantly different (*p*-value = 0.0001 for the Kruskal–Wallis test). In descending order: strain 717, the reference strain, and strain 647. Each strain was statistically different from the others (*p*-value < 0.05 for the Wilcoxon test). 

#### 3.2.2. For *M. intracellulare*

At 48 h: The viability of the three strains was significantly different (*p*-value = 0.0004 for the Kruskal–Wallis test). In descending order: the reference strain, strain 505, and strain 657. The viability of strain 657 was significantly lower than the rest of the strains (*p*-value < 0.05). There were no significant differences between the reference strain and strain 505 (*p*-value = 0.1242). The covered surface of the strains was significantly different (*p*-value = 0.0001 for the Kruskal–Wallis test). In descending order: strain 505, the reference strain, and strain 657. Each strain was statistically different from the others (*p*-value < 0.05 for the Wilcoxon test). The autofluorescence of the three strains was significantly different (*p*-value = 0.0001 for the Kruskal–Wallis test). In descending order: strain 657, the reference strain, and strain 505. Each strain was statistically different from the others (*p*-value < 0.0001 for the Wilcoxon test). The biofilm height of the three strains was significantly different (*p*-value = 0.0001 for the Kruskal–Wallis test). In descending order: strain 657, the reference strain, and strain 505. Each strain was statistically different from the others (*p*-value < 0.001 for the Wilcoxon test).

At 120 h: The viability of the three strains was significantly different (*p*-value = 0.0001 for the Kruskal–Wallis test). In descending order: the reference strain, strain 505, and strain 657. The viability of the 657 strain was significantly higher than the reference strains and 505 (*p*-value < 0.0001 for the Wilcoxon test). There were no significant differences between strain 505 and the reference strain (*p*-value = 0.0863). The surface area covered by the reference and 505 strains was similar (*p*-value = 0.0001 for the Wilcoxon test). In descending order: strain 505, the reference strain, and strain 657. Each strain was statistically different from the others (*p*-value < 0.01 for the Wilcoxon test). The autofluorescence of the three strains was significantly different (*p*-value = 0.0001 for the Kruskal–Wallis test). In descending order: strain 657, strain 505, and the reference strain. Each strain was statistically different from the others (*p*-value < 0.01 for the Wilcoxon test). The biofilm height of the three strains was significantly different (*p*-value = 0.0001 for the Kruskal–Wallis test). In descending order: strain 657, the reference strain, and strain 505. Each strain was statistically different from the others (*p*-value < 0.01 for the Wilcoxon test).

At 192 h: The viability of the three strains was statistically different (*p*-value = 0.0001 for the Kruskal–Wallis test). In descending order: the reference strain, strain 505, and strain 657. The viability of strain 657 was significantly lower than the other two strains (*p*-value < 0.0001 for the Wilcoxon test). There were no significant differences between strain 505 and the reference strain (*p*-value = 0.1354). The surface area covered by the three strains was significantly different (*p*-value = 0.0001 for the Kruskal–Wallis test). In descending order: strain 505, the reference strain, and strain 657. Each strain was statistically different from the others (*p*-value < 0.001 for the Wilcoxon test). The autofluorescence of the three strains was significantly different (*p*-value = 0.0001 for the Kruskal–Wallis test). In descending order: strain 657, the reference strain, and strain 505. Each strain was statistically different from the others (*p*-value < 0.001 for the Wilcoxon test). The biofilm height of the three strains was significantly different (*p*-value = 0.0001 for the Kruskal–Wallis test). In descending order: strain 657, strain 505, and the reference strain. The biofilm height of the reference strain was significantly lower than the other two strains (*p*-value < 0.0001 for the Wilcoxon test). There were no significant differences between strain 657 and strain 505 (*p*-value = 0.11896).

At 264 h: The viability of the three strains was significantly different (*p*-value = 0.0001 for the Kruskal–Wallis test). In descending order: the reference strain, strain 505, and strain 657. Each strain was statistically different from the others (*p*-value < 0.001 for the Wilcoxon test). The covered surface was significantly different (*p*-value = 0.0001 for the Kruskal–Wallis test). In descending order: strain 505, the reference strain, and strain 657. Each strain was statistically different from the others (*p*-value < 0.001 for the Wilcoxon test). The autofluorescence of the three strains was significantly different (*p*-value = 0.0001 for the Kruskal–Wallis test). In descending order: strain 657, the reference strain, and strain 505. Each strain was statistically different from the others (*p*-value < 0.001 for the Wilcoxon test). The biofilm height of the three strains was significantly different (*p*-value = 0.0001 for the Kruskal–Wallis test). In descending order: strain 657, strain 505, and the reference strain. Each strain was statistically different from the others (*p*-value < 0.001 for the Wilcoxon test).

#### 3.2.3. For *M. chimaera*

At 48 h: The viability of the three strains was significantly different (*p*-value = 0.0001 for the Kruskal–Wallis test). In descending order: strain 655, the reference strain, and strain 575. The viability of strain 655 was significantly higher than the rest of the strains (*p*-value < 0.0001). There were no significant differences between the reference strain and strain 575 (*p*-value = 0.2254). The covered surface of the strains was significantly different (*p*-value = 0.0001 for the Kruskal–Wallis test). The surface area covered by strain 655 was significantly greater than the reference strains and 575 (*p*-value < 0.05 for the Wilcoxon test). There were no significant differences between the reference strain and strain 575 (*p*-value = 0.6256). The autofluorescence of the three strains was significantly different (*p*-value = 0.0001 for the Kruskal–Wallis test). In descending order: the reference strains, strain 575, and strain 655. Each strain was statistically different from the others (*p*-value < 0.01 for the Wilcoxon test). The biofilm height of the three strains was not significantly different (*p*-value = 0.963 for the Kruskal–Wallis test).

At 120 h: The viability of the three strains was significantly different (*p*-value = 0.0001 for the Kruskal–Wallis test). In descending order: strain 575, the reference strain, and strain 655. Each strain was statistically different from the others (*p*-value < 0.001 for the Wilcoxon test). The surface area covered by the reference and 575 strains was similar (*p*-value = 0.0001 for the Wilcoxon test). The surface area covered by strain 655 was significantly greater than the reference strain and 575 (*p*-value < 0.0001 for the Wilcoxon test). There were no significant differences between strain 575 and the reference strain (*p*-value = 0.1184). The autofluorescence of the three strains was significantly different (*p*-value = 0.0001 for the Kruskal–Wallis test). In descending order: the reference strains, strain 575, and strain 655. The autofluorescence of the reference strain was significantly higher than that of strain 655 (*p*-value = 0.0232 for the Wilcoxon test). There were no significant differences between strain 575 and strain 655 or between strain 575 and the reference strain (*p*-value > 0.15). The biofilm height of the three strains was significantly different (*p*-value = 0.0001 for the Kruskal–Wallis test). The biofilm height of strain 655 was significantly higher than the other two strains (*p*-value < 0.0001 for the Wilcoxon test). There were no significant differences between strain 575 and the reference strain (*p*-value = 0.6492).

At 192 h: The viability of the three strains was statistically different (*p*-value = 0.0001 for the Kruskal–Wallis test). In descending order: strain 655, strain 575, and the reference strain. Each strain was statistically different from the others (*p*-value < 0.0001 for the Wilcoxon test). The surface area covered by the three strains was significantly different (*p*-value = 0.0001 for the Kruskal–Wallis test). The surface covered by biofilm of the reference strain was significantly lower than the other two strains (*p*-value < 0.0001 for the Wilcoxon test). There were no significant differences between strain 575 and strain 655 (*p*-value = 0.9411). The autofluorescence of the three strains was significantly different (*p*-value = 0.0475 for the Kruskal–Wallis test). The autofluorescence of the reference strain was significantly higher than that of strain 655 (*p*-value = 0.0232 for the Wilcoxon test). There were no significant differences between strain 575 and strain 655 or between strain 575 and the reference strain (*p*-value > 0.15). The biofilm height of the three strains was significantly different (*p*-value = 0.0001 for the Kruskal–Wallis test). The biofilm height of strain 655 was significantly lower than the other two strains (*p*-value < 0.001 for the Wilcoxon test). There were no significant differences between strain 655 and the reference strain (*p*-value = 0.1958).

At 264 h: The viability of the three strains was significantly different (*p*-value = 0.0031 for the Kruskal–Wallis test). In descending order: strain 655, strain 575, and the reference strain. The viability of strain 655 was significantly higher than the other two strains studied (*p*-value < 0.05 for the Wilcoxon test). There were no significant differences between strain 575 and the reference strain (*p*-value = 0.2544). The surface area covered by the reference and 575 strains was similar (*p*-value = 0.0001 for the Wilcoxon test). The surface area covered by the reference strain was significantly smaller than the other two strains studied (*p*-value < 0.0001 for the Wilcoxon test). There were no significant differences between strain 575 and strain 655 (*p*-value = 0.9411). The autofluorescence of the three strains was not significantly different (*p*-value = 0.5166 for the Kruskal–Wallis test). The biofilm height of the three strains was significantly different (*p*-value = 0.0001 for the Kruskal–Wallis test). The height of strain 575 was significantly greater than the other two strains studied (*p*-value < 0.0001 for the Wilcoxon test). There were no significant differences between strain 575 and strain 655 (*p*-value = 0.1858).

### 3.3. Antimicrobial Susceptibility of Planktonic and Sessile Bacteria 

The results of susceptibility testing of all strains appear in Table 1.

### 3.4. Effect of Clarithromycin in Biofilm Ultrastructure 

**Figure 3:**
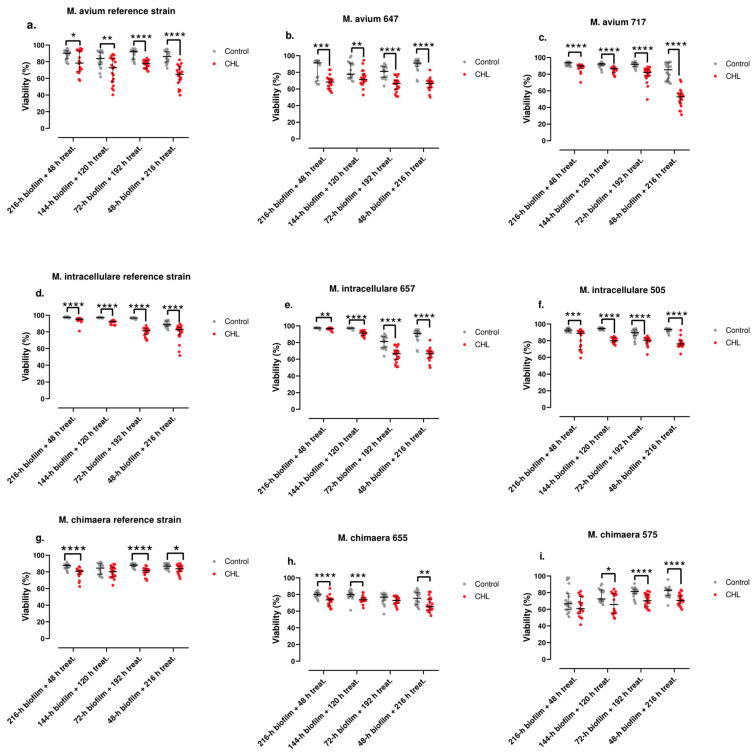
Biofilm viability treated with clarithromycin for *M. avium* (**a**–**c**), *M. intracellulare* (**d**–**f**), and *M. chimaera* (**g**–**i**) clinical strains. The bars represent the interquartile range. *: *p*-value < 0.05, **: *p*-value < 0.01, ***: *p*-value < 0.001, and ****: *p*-value < 0.0001 for Wilcoxon test.

The results of this study are shown in Figure 3.

The viability of the bacteria contained in the biofilm of *M. avium* strain 717 showed a strong negative correlation with treatment time (ρ = −0.8211; *p*-value < 0.0001). The viability of the bacteria contained in the biofilm of the *M. avium* reference strain showed a weak negative correlation with treatment time (ρ = −0.3239; *p*-value = 0.0033). The viability of the bacteria contained in the biofilm of *M. avium* strain 647 did not show a clear correlation with treatment time (*p*-value = 0.0714). The viability of the bacteria contained in the biofilm of the *M. intracellulare* reference strain showed a very strong positive correlation with treatment time (ρ = −0.8090; *p*-value < 0.0001). The viability of the bacteria contained in the biofilm of *M. intracellulare* strain 505 showed a weak negative correlation with treatment time (ρ = −0.3045; *p*-value = 0.0060). The viability of the bacteria contained in the biofilm of *M. intracellulare* strain 657 showed a very strong negative correlation with treatment time (ρ = −0.9281; *p*-value < 0.0001). The viability of the bacteria contained in the biofilm of the *M. chimaera* reference strain showed a weak positive correlation with treatment time (ρ = 0.2783; *p*-value = 0.0124). The viability of the bacteria contained in the biofilm of *M. chimaera* strain 575 showed a weak positive correlation with treatment time (ρ = 0.2871; *p*-value = 0.0103). The viability of the bacteria contained in the biofilm of *M. chimaera* strain 655 showed a weak negative correlation with treatment time (ρ = −0.2271; *p*-value = 0.0428). 

## 4. Discussion 

The *M. avium* complex is a group of non-tuberculous mycobacteria that includes mainly three species (*M. avium*, *M. intracellulare*, and *M. chimaera*) that have been described as human pathogens [18,19], especially in the respiratory tract in patients with underlying diseases, as well as disseminated diseases in immunosuppressed patients, especially AIDs patients [20]. 

The infections caused by these organisms are difficult to diagnose because in some cases they can appear as colonizations or contaminations from environmental sources. For this purpose, several guidelines have been published that include specific criteria for the diagnosis of *M. avium* complex infections. Many of these infections (especially lung ones) have been considered as biofilm-related ones. The ability to develop biofilms by these mycobacteria was proved several years ago, and this property is now considered an essential pathogenic factor for these infections [11,21,22,23]. Moreover, because sessile bacteria appear to be more resistant to antibiotics than planktonic ones, therapy for these patients must be influenced by this finding [24], and prolonged treatment with a combination of different antimicrobials is mandatory. However, despite these recommendations, in some cases treatment failures appear, and these failures could be related to the existence of biofilms in the patient [20]. Previous studies have shown that the *M. avium* complex can form biofilm in vitro [9,10], and some techniques have been tested, aiming at minimizing the colonization and subsequent development of biofilms in biomedical surfaces [25]. However, most of these studies have been performed with collection strains. In this study, we tested clinical strains not only of different species but of different clinical relevance, so our results are of special interest because of the potential differences that can appear.

The main aim of this study was to characterize biofilm formation by using Nile Red© stains and Live/Dead BacLight© stains with a previously described protocol to analyze several parameters (the % of the covered surface, thickness, viability, and presence of autofluorescence) with and without antibiotics to determine their relative importance in treatment resistance and looking to see if there are species-specific patterns. In our study, we found many differences in the different parameters between the different species. However, the obtained values do have neither a species-specific nor a clinical significance-specific pattern, a similar result than appear in other studies with other characteristics [26]. Other studies relating to biofilm formation for *M. avium* and *M. intracellulare* clinical strains found *M. avium* to produce stronger biofilm than *M. intracellulare* [7], but we have not detected this difference when all the strains of the same species were analyzed together. Although more studies are needed to understand MAC biofilm formation to determine more specific treatments for patients with MAC-related infections, we can conclude that biofilm formation is strain-dependent, as no clear differences between species can be detected.

Experiments were also carried out on the nine clinical isolates treated with clarithromycin by using the Live/Dead BacLight© stain to differentiate the live and dead cells from the biofilm and also determine the MIC, MBC, and MBIC of these strains. In these experiments, again we do not find any common pattern among the different isolates, while most isolates showed higher activity against younger biofilms + prolonged treatment than against older ones + short treatment, with only two strains (both of them *M. chimaera*, DSM756 and strain 575) showing only a weak positive correlation with time. In this study, it is difficult to know with this methodology if the effect is due to the treatment time or the age of the biofilm, but the percentage of the biofilm viability of the strains evaluated was not less than 40% at best, even with the highest time of the clarithromycin treatment, which suggests that biofilm is an extremely important parameter in the resistance of these organisms and the need for prolonged treatments, as recommended in the guidelines [4]. The high degree of resistance against clarithromycin in the static studies suggests that the age of the biofilm could be the parameter of the highest importance in the results. However, the finding of almost a lack of effect in two of the *M. chimaera* strains at different times suggests that other parameters probably influence the effect of the antibiotic in the biofilms, and the differences between this species and the other ones from the complex need further studies with more clinical strains. *Mycobacterium avium* complex strains, when appearing as biofilms attached to other surfaces, show more resistance against clarithromycin than in conventional studies using planktonic cells against clarithromycin and also rifampin [27]. This increased resistance has also been demonstrated in other species of mycobacteria by using a similar methodology [13] and could be one explanation for the problems in the treatment of the infections caused by these organisms.

In conclusion, the biofilm-forming ability in *M. avium* complexes appears to be a strain-specific property that does not depend on the species or any other characteristic. The effect of antibiotic therapy with clarithromycin is also strain-specific, and the treatment of older biofilms seems to have less effect than that of the treatment of younger biofilms.

## Figures and Tables

**Table 1:** Susceptibility testing data for planktonic and sessile bacteria.

Strain	MIC (µg/mL)	MBC (µg/mL)	MBIC (µg/mL)
*M. avium* CECT 670	4	<256	16
*M. avium* 717	8	8	8
*M. avium* 647	≤1	≤1	≤1
*M. intracellulare* 669	≤1	32	≤1
*M. intracellulare* 505	≤1	≤1	≤1
*M. intracellulare* 657	≤1	32	≤1
*M. chimaera* DSM 756	≤1	32	16
*M. chimaera* 575	≤1	8	≤1
*M. chimaera* 655	8	16	≤1

MIC: Minimal Inhibitory Concentration; MBC: Minimal Bactericidal Concentration; MBIC: Minimal Biofilm Inhibitory Concentration.

## Data Availability

Data are contained within the article.
